# Application of mosaicism ratio to multifetal gestations

**DOI:** 10.1371/journal.pone.0248467

**Published:** 2021-03-12

**Authors:** Jill Rafalko, Samantha Caldwell, Erica Soster, Eyad Almasri, Graham McLennan, Tong Liu, Vivian Weinblatt, Philip Cacheris, Ron McCullough

**Affiliations:** Laboratory Corporation of America, La Jolla, California, United States of America; Clinic Hospital of Zaragoza, SPAIN

## Abstract

Mosaicism ratio, or MR, is a laboratory metric that can be calculated using massively parallel sequencing data from cell-free DNA (cfDNA) screening. MR compares the amount of cfDNA present from a particular chromosome or chromosomal region to the overall fetal fraction of the specimen. In singleton gestations, MR may be used to refine the positive predictive value of an abnormal cfDNA screening result by identifying cases that could be impacted by various biological factors, such as placental mosaicism or prior co-twin demise. The current study was designed to examine the behavior of mosaicism ratio (MR) in multifetal gestations. Multifetal cfDNA specimens with positive results for trisomies 21, 18, or 13 and confirmed diagnostic outcomes were compiled to examine MR of the aneuploid chromosome based on the number of affected fetuses/placentas. A second multifetal cohort was assembled to analyze the MR of the Y chromosome in cases with at least one male fetus. For aneuploid cases, the average MR of affected singletons (used as a biological proxy for two affected twins) was significantly higher than the average MR for twins in which one fetus was affected. The average MR of the aneuploid chromosome for one affected twin was 52%, 42%, and 48% of that of singleton gestations for trisomy 21, 18, and 13 cases, respectively. MR cutoffs of 0.7 for trisomy 21, and 0.5 for trisomies 18 and 13 may help predict whether one versus both twins are affected with aneuploidy when clinical concern arises. For male cases, the Y MR of XX/XY gestations was 48% of the Y MR for XY/XY gestations. Using a Y MR cutoff of 0.8 allowed determination of XX/XY versus XY/XY gestations with 92.3–94.9% accuracy. Based on the data presented, MR may have utility in the analysis and interpretation of cfDNA data from multifetal gestations.

## Introduction

In singleton gestations, mosaicism ratio is a laboratory metric calculated in the event of a positive cell-free DNA (cfDNA) screen. It is derived by dividing the fraction of cfDNA affected by aneuploidy by the overall fetal fraction of the specimen. This metric may help identify results more likely to be discordant with the genetic status of the fetus due to mosaicism, co-twin demise, or other biological factors. By extension, this metric may be helpful in refining the positive predictive value (PPV) associated with the result [1].

The mosaicism ratio (MR) metric is not exclusive to singleton gestations. In multifetal pregnancies, when results indicate an overrepresentation of cfDNA suggestive of aneuploidy, MR may be useful in predicting whether one or more fetuses are affected. Additionally, when Y chromosome material is detected in a multifetal gestation, the MR associated with the Y chromosome may be useful in predicting whether one or more fetuses are male. Prior studies have focused on similar data metrics to develop a fetal sex prediction model for twin gestations [2, 3].

The current study examines the behavior of MR in multifetal gestations in two contexts: MR of the ‘affected chromosome’ in cases of confirmed aneuploidy, and MR associated with the Y chromosome in cases where Y material is detected and the chromosomal sex is known for all fetuses. Results from cfDNA screening were matched to diagnostic outcomes and the data were used to assess the utility of MR in multifetal pregnancies.

## Materials and methods

For the current study, cfDNA samples were compiled from multifetal gestations from two sources: 1) Clinical specimens submitted for the MaterniT^®^21 PLUS test from September 2013 through February 2020; and, 2) Research specimens collected under an IRB-approved clinical study (NCT01429389).

### Clinical specimens

**Fig 1:**
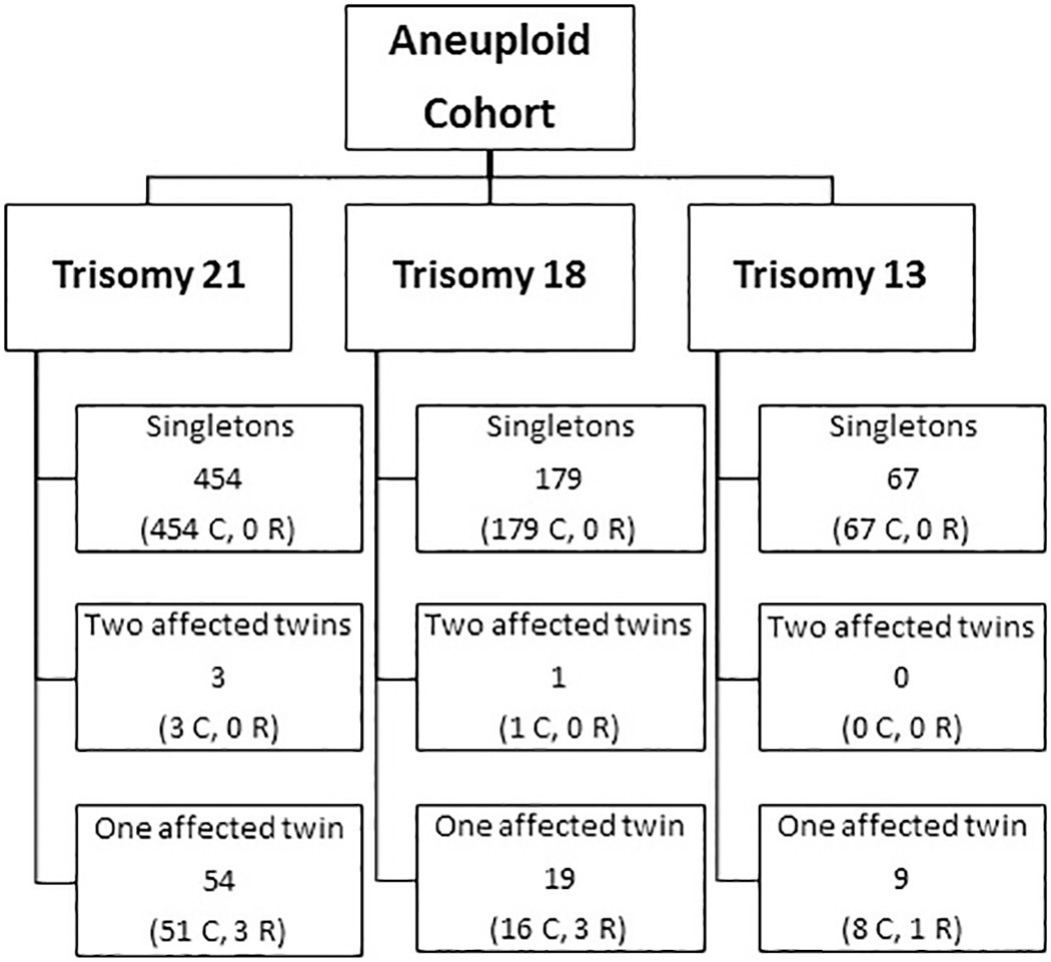
[Aneuploid Cohort: Clinical + Research specimens] composition of specimens included in the Aneuploid Cohort. C = Clinical specimens; R = Research specimens.

**Fig 2:**
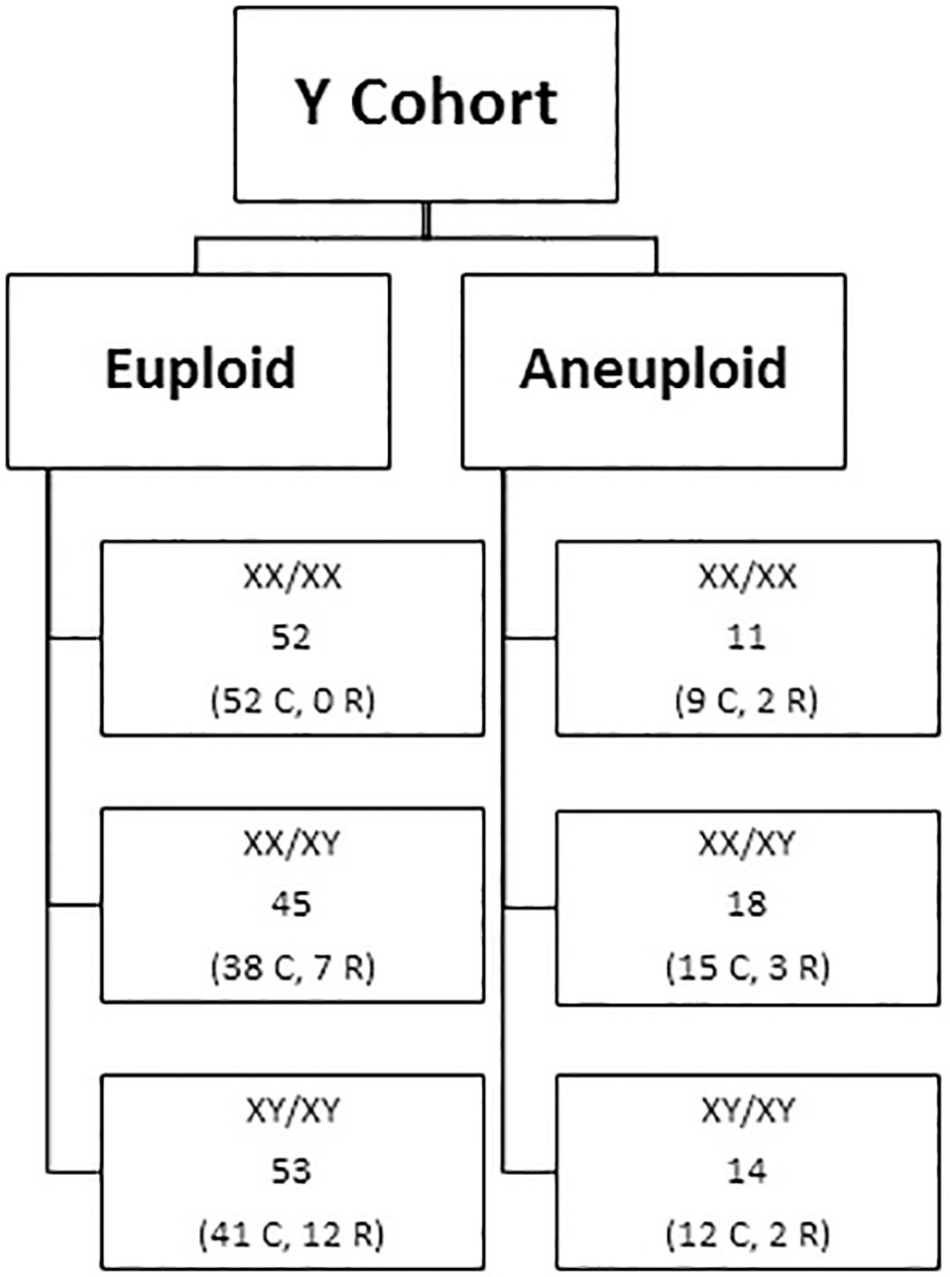
[Y Cohort: Clinical + Research specimens] composition of specimens included in the Y Cohort. C = Clinical specimens; R = Research specimens.

Maternal blood samples from multifetal pregnancies submitted for cfDNA screening during the course of routine clinical care from September 2013 through February 2020 were compiled. Blood samples were shipped in the provided commercial kits at ambient temperature to the CAP accredited CLIA laboratory and processed upon arrival. Multifetal gestations were identified by a fetal number of “2” or greater as indicated by the ordering provider on the cfDNA test requisition form. Details regarding the number and type of clinical specimens analyzed can be found in Figs 1 and 2.

### Research specimens

Research specimens were collected under the multi-center clinical study SQNM-T21-107 (NCT01429389). Each participating site obtained local IRB approval for the protocol and informed consent form prior to enrolling subjects. The process of consolidation and comparison of data was approved by Aspire-IRB under clinical protocol SCMM-RND-402 (NCT04364503). Maternal blood samples were collected from women with multifetal gestations prior to undergoing prenatal diagnostic testing via chorionic villus sampling or amniocentesis after providing written informed consent to participate under the IRB approved protocol SQNM-T21-107 (NCT01429389). These pregnancies were identified at increased risk for aneuploidy due to advanced maternal age, personal or family history, abnormal serum screening, or ultrasound anomalies. Whole blood samples were shipped at ambient temperature to Sequenom Clinical Affairs (San Diego, California, USA) for processing to plasma as previously described. Samples were identified from collection through processing by a unique 5-digit identifier, were completely devoid of any patient identifying information, and were stored for future use at ≤-70°C.

The research specimens were removed from the freezer, assigned a specimen identification number, and tested in the same manner as specimens being submitted for routine clinical testing using massively parallel sequencing. The diagnostic outcomes for these research specimens were maintained by Sequenom Clinical Affairs in a password protected, limited access database and were blinded to all laboratory personnel involved in processing, testing, and reporting of the specimen.

Once cfDNA results were reported, the authors matched the sample’s cfDNA result with the previously-documented diagnostic result based on the sample’s 5-digit identifier. Details regarding the number and type of research specimens analyzed can be found in Figs 1 and 2.

### cfDNA analysis and calculation of mosaicism ratio

Maternal blood samples were subjected to DNA extraction, library preparation, and genome-wide massively parallel sequencing, as previously described [4]. Fetal fraction was assessed for each specimen, as described by Kim, et al. [5].

As previously described, in samples with a detected copy number variant (CNV) involving a whole chromosome or subchromosomal region, an ‘affected fraction’ can be determined by calculating the fraction of cfDNA required to generate the observed change of sequencing counts in the CNV region. Once an ‘affected fraction’ is derived, mosaicism ratio can be calculated. As previously described, MR is derived by dividing the ‘affected fraction’ estimated for the aberrant chromosome or chromosomal segment over the fetal fraction estimated for all chromosomes [1, 6, 7].

In the case of a non-mosaic CNV affecting the entire placenta of a monochorionic twin gestation, or affecting both placentas of a dichorionic twin gestation, the affected fraction should be approximately equivalent to overall fetal fraction, and MR is expected to be roughly 1.0. In the event of a non-mosaic CNV affecting one placenta of a dichorionic twin pregnancy, the affected fraction is, theoretically, expected to be approximately half of the overall fetal fraction. This is only considered an approximation, as the two placentas in a twin gestation may contribute unequal amounts of cfDNA during pregnancy [8]. A depressed MR suggests that there is less aneuploid cfDNA contribution than there is fetal fraction, which may be indicative of one affected fetus in a multifetal gestation, placental mosaicism or other biological phenomena, such as prior demise of an additional fetus.

### Diagnostic outcomes

As noted above, for the research specimens, diagnostic outcomes from karyotype and/or microarray were provided by the submitting clinician and these outcomes were blinded to the laboratory personnel that processed, tested, and reported the specimens.

For the clinical specimens, diagnostic outcomes were obtained from two sources. First, outcome information from *ad hoc* feedback was collected, when available, from the ordering provider. Outcomes based on clinician feedback were used as the source of diagnostic information for 21 of the true positive twin cases in the Aneuploid Cohort (described below). Second, positive cfDNA samples were cross-referenced with results of cytogenetic and SNP microarray diagnostic testing submitted to Labcorp from chorionic villus, amniocentesis, postnatal blood, and product of conception specimens during a corresponding timeframe. Cases with mosaic diagnostic results, confirming the aneuploidy detected by cfDNA, were included. The process of consolidation and comparison of data was approved by Aspire-IRB under clinical protocol SCMM-RND-402 (NCT04364503).

For a cfDNA sample to be considered a match to a cytogenetic and/or microarray specimen, the diagnostic and screening results were required to have identical patient identifiers (name and date of birth), and the collection date for the diagnostic test had to be within 90 days of the patient’s cfDNA screening date. When multiple diagnostic results (e.g. cytogenetic and microarray results, or CVS and amniocentesis results) were available for the same patient, results were combined under one final characterization.

For purposes of this study, diagnostic results were required for each fetus of the twin pair, unless the pregnancy was explicitly noted to be monochorionic/identical, or the indication for diagnostic amniocentesis was twin-twin transfusion syndrome (TTTS), a condition only present in monochorionic twins. Cases of known co-twin demise and cases with only one diagnostic result (with no documentation of monochorionic twins) were excluded.

### Cohorts analyzed

Two cohorts were assembled and analyzed from the research and clinical specimens: an ‘Aneuploid Cohort’ and a ‘Y Cohort’.

The first cohort, denoted the “Aneuploid Cohort”, was compiled to examine MR of the aneuploid chromosome based on the number of affected fetuses/placentas. Three groups were analyzed in this cohort. The first group included twin cases for which the cfDNA results were positive for trisomy 21, 18, or 13 and diagnostic testing confirmed the predicted aneuploidy in one fetus. The second group was comprised of twin gestations in which both fetuses were confirmed to have the same aneuploidy (trisomy 21, 18, or 13). There were only four clinical cases identified for this cohort: one set of mixed-sex, dichorionic twins, both with trisomy 21; two cases of monochorionic twins with trisomy 21; and, one case of monochorionic twins with trisomy 18. There are a small number of cases in this group because information regarding chorionicity is not routinely elicited from the ordering provider on the laboratory’s test requisition form. Given the limited number of twin cases with two affected fetuses, a third group of singletons with confirmed positive results for trisomy 21, 18, or 13 was also assembled. This singleton group was assembled to biologically mimic the scenario of affected monochorionic twins. Both singleton gestations and monochorionic twins involve cfDNA analysis from a single placenta, and prior studies have demonstrated that monochorionic twin pregnancies behave similarly to singletons in the context of cfDNA screening [3, 9]. By extension, in the rare circumstance that both placentas of a dichorionic pregnancy are affected with the same, non-mosaic aneuploidy (i.e. from independent nondisjunction events), the two placentas would, presumably, be contributing aneuploid cfDNA at a level proportional to the fetal fraction, similar to how a non-mosaic aneuploidy would behave when a single placenta is affected.

The second cohort, denoted the “Y Cohort”, was assembled to analyze the MR behavior of the Y chromosome in twin gestations where the chromosomal sex of both fetuses was known from karyotype and/or microarray and at least one fetus was male.

An additional group of five triplet gestations with positive cfDNA screening and partial or full diagnostic outcome information were compiled from the clinical samples, along with three euploid triplet cases and one euploid quadruplet case from the research specimens.

Comparison of mean mosaicism ratios was performed using a two-sided t-test. Confidence intervals were calculated via VassarStats website [10].

## Results

### Research specimens

Of the 31 research specimens tested prospectively, 30 samples were reportable. There was one non-reportable result due to low fetal fraction from a twin pregnancy in which one fetus was affected with trisomy 21. Aside from this one non-reportable result, all of the aneuploid specimens correctly reported as positive for the trisomy identified by diagnostic testing (i.e. three trisomy 21 cases, three trisomy 18 cases, and one trisomy 13 case). Fetal sex calls regarding presence or absence of Y chromosome material were correct for all aneuploid cases. All euploid research specimens analyzed for Y MR (19 twins, 3 triplets, one set of quadruplets) appropriately reported with negative, male results.

### Aneuploid Cohort

For trisomy 21, there was no significant difference between the mean mosaicism ratio (MR) in singletons (1.13 ± 0.28, n = 454) when compared to twins with two affected fetuses (1.22 ± 0.12, n = 3), p = .58. When only one fetus of the twin pair was affected with trisomy 21, the mean MR was 0.59 ± 0.20 (n = 54), which was significantly lower (p < .001) than the mean MR of the affected singleton group, as well as the cases with two affected fetuses (Table 1; S1 Fig).

**Table 1:** [Aneuploid Cohort: Clinical + Research specimens] comparison of aneuploid chromosome mosaicism ratio among affected singletons, two affected twins, and one affected twin, by aneuploidy.

	Mean	Range	Interquartile Range
**Trisomy 21**			
Singletons (n = 454)	1.13 ± 0.28	0.32–2.53	0.96–1.28
Two affected twins (n = 3)	1.22 ± 0.12	1.13–1.35	1.13–1.35
One affected twin (n = 54)	0.59 ± 0.20	0.20–1.07	0.46–0.69
**Trisomy 18**			
Singletons (n = 179)	0.90 ± 0.29	0.10–2.00	0.69–1.10
Two affected twins (n = 1)	0.73	n/a	n/a
One affected twin (n = 19)	0.38 ± 0.12	0.18–0.70	0.28–0.46
**Trisomy 13**			
Singletons (n = 67)	0.89 ± 0.29	0.19–1.53	0.66–1.13
Two affected twins (n = 0)	n/a	n/a	n/a
One affected twin (n = 9)	0.43 ± 0.18	0.24–0.88	0.34–0.46

For trisomy 18, the mean MR in singletons was 0.90 ± 0.29 (n = 179). There was one case of monochorionic twins affected with trisomy 18, with an MR of 0.73. In cases where only one fetus of the twin pair was affected with trisomy 18, the mean MR was 0.38 ± 0.12 (n = 19), which is significantly lower (p < .001) than the mean MR of the affected singleton group (Table 1; S1 Fig).

For trisomy 13, the mean MR in singletons was 0.89 ± 0.29 (n- = 67). There were no cases identified in which both twins were affected with trisomy 13. In cases where only one fetus of the twin pair was affected with trisomy 13, the mean MR was 0.43 ± 0.18 (n = 9), which is significantly lower (p < .001) than the mean MR of the affected singleton group (Table 1; S1 Fig).

Analysis of the distribution of aneuploid MRs by trisomy can be seen in Table 2.

**Table 2:** [Aneuploid Cohort: Clinical + Research specimens] distribution of aneuploid MR by trisomy.

	Trisomy 21		Trisomy 18		Trisomy 13	
Mosaicism ratio by 0.1 range	One affected twin	Affected singletons	One affected twin	Affected singletons	One affected twin	Affected singletons
**<0.3**	3	2	5	5	1	1
**0.3–0.39**	7	0	5	2	5	1
**0.4–0.49**	7	0	6	5	1	1
**0.5–0.59**	8	7	1	11	1	6
**0.6–0.69**	17	11	1	25	0	12
**0.7–0.79**	3	24	1	18	0	8
**0.8–0.89**	3	46	0	17	1	6
**0.9–0.99**	3	50	0	30	0	8
**>1.0**	3	314	0	66	0	24
**Total**	**54**	**454**	**19**	**179**	**9**	**67**

Shading indicates the proposed threshold (0.7 for trisomy 21, 0.5 for trisomy 18/13) for prediction of one versus both fetuses affected with aneuploidy.

### Y Cohort

The mean Y MR for euploid cases was 0.51 ± 0.15 for XX/XY twins (n = 45) and 1.04 ± 0.18 for XY/XY twins (n = 53). The mean Y MR for aneuploid cases was 0.54 ± 0.25 for XX/XY twins (n = 18) and 1.11 ± 0.27 for XY/XY twins (n = 14). There was no significant difference between the mean Y MRs of the euploid and the aneuploid cases (p = 0.56 for XX/XY cases and p = 0.28 for XY/XY cases).

For the overall cohort, including euploid and aneuploid cases from both clinical and research specimens (n = 130), the mean Y MR for XX/XY cases was 0.51 ± 0.19 (n = 63), and the mean Y MR for XY/XY cases was 1.06 ± 0.20 (n = 67) (Table 3; S2 Fig).

**Table 3:** [Y Cohort: Clinical + Research specimens] comparison of Y chromosome mosaicism ratio between XX/XY and XY/XY twins in euploid, aneuploid, and combined cases.

	Mean	Range	Interquartile Range
**Euploid cases**			
XX/XY (n = 45)	0.51 ± 0.15	0.19–0.90	0.40–0.63
XY/XY (n = 53)	1.04 ± 0.18	0.60–1.40	0.93–1.16
**Aneuploid cases**			
XX/XY (n = 18)	0.54 ± 0.25	0.19–0.95	0.28–0.80
XY/XY (n = 14)	1.11 ± 0.27	0.58–1.46	0.96–1.31
**Combined (euploid + aneuploid cases)**			
XX/XY (n = 63)	0.51 ± 0.19	0.19–0.95	0.40–0.64
XY/XY (n = 67)	1.06 ± 0.20	0.58–1.46	0.93–1.20

It should be noted that 52 euploid and 11 aneuploid twin gestations with two female fetuses were also examined, but all Y MR values were essentially zero, with a mean Y MR of 0.00 ± 0.01. The probability of an XX/XX outcome when Y chromosome material was absent from a cfDNA specimen was 100% in this study population.

Using the Y MR data, various models were tested to predict the number of male fetuses present in a twin gestation where Y material is detected. Of these models, the best accuracy for fetal sex determination came from a one-dimensional model, using a single value cutoff. The model predicts that when Y material is detected and Y MR is <0.8, the most likely outcome is XX/XY fetuses, and when Y MR is >0.8, the most likely outcome is XY/XY fetuses. The accuracy of using a 0.8 cutoff was 95.9% for euploid cases (n = 98) (Table 4). Similar accuracy (94.9%) was obtained when the cutoff was set between 0.7 and 0.8, suggesting that the model/cutoff is robust (Table 5). If aneuploid cases were included, the accuracy of using a 0.8 cutoff would decrease to 92.3% (n = 130) (Table 6).

**Table 4:** [Y Cohort: Clinical + Research specimens] probability of XX/XY versus XY/XY outcome based on Y MR when Y chromosome material is detected. Includes only euploid cases.

Distribution of Y MRs by MR range and known fetal sex combinations in euploid twins						
MR range	XX/XY	XY/XY	# of cases	MR range	Probability of XX/XY outcome (95% CI)	Probability of XY/XY outcome (95% CI)
**<0.8**	43	2	45	**<0.8**	95.6% (83.6–99.2)	4.4% (0.7–16.4)
**≥0.8**	2	51	53	**≥0.8**	3.8% (0.7–14.1)	96.2% (85.9–99.4)
**Total cases**	45	53	98			
**Accuracy**	**(TP+TN)/(TP+TN+FP+FN) = 0.959 = 95.9%**					

**Table 5:** Accuracy of twin fetal sex prediction (XX/XY vs. XY/XY outcome when Y material is detected) in euploid gestations at various Y MR cutoffs. Analysis based on Y MRs from 98 euploid samples.

Y MR cutoffs	Accuracy
0.5	0.795918
0.55	0.816327
0.6	0.867347
0.65	0.908163
**0.7**	**0.94898**
**0.75**	**0.94898**
**0.8**	**0.959184**
0.85	0.918367
0.9	0.897959
0.95	0.816327
1	0.77551

**Table 6:** [Y Cohort: Clinical + Research specimens] probability of XX/XY versus XY/XY outcome based on Y MR when Y chromosome material is detected. Includes euploid and aneuploid cases.

Distribution of Y MRs by MR range and known fetal sex combinations in euploid + aneuploid twins						
MR range	XX/XY	XY/XY	# of cases	MR range	Probability of XX/XY outcome (95% CI)	Probability of XY/XY outcome (95% CI)
**<0.8**	57	4	61	**<0.8**	93.4% (83.3–97.9)	6.6% (2.1–16.8)
**≥0.8**	6	63	69	**≥0.8**	8.7% (3.6–18.6)	91.3% (81.4–96.4)
**Total cases**	63	67	130			
**Accuracy**	**(TP+TN)/(TP+TN+FP+FN) = 0.923 = 92.3%**					

### Triplets and quadruplets

In addition to the twin cases described above, five triplet cases with positive cfDNA results and at least partial clinical or diagnostic outcome information were identified from clinical specimens. A summary of the mosaicism ratios (for the aneuploid chromosome and the Y chromosome) and clinical information is shown in Table 7.

**Table 7:** [Aneuploid Cohort: Clinical specimens] triplet cases with positive cfDNA results and outcome information.

cfDNA result	Gestational age at draw	Fetal fraction of specimen	MR of aneuploid chromosome	Y MR	Clinical information
**Positive trisomy 21; Y detected**	9 weeks	11.1%	0.40	0.63	Amniocentesis:
					47,XY,+21
					46,XX
					46,XY
**Positive trisomy 21; Y not detected**	10 weeks	10.0%	0.62	0	CVS:
					46,XX
					47,XX,+21
					47,XX,+21
**Positive trisomy 21; Y not detected**	9 weeks	9.4%	0.77	0	Triplet A: nuchal edema, possible AV canal defect (selective reduction, no diagnostic studies)
					Triplet B: echogenic focus, IUGR—trisomy 21 diagnosed postnatally (female)
					Triplet C: normal at birth (female)
**Positive trisomy 21; Y detected**	13 weeks	11.0%	0.33	0.19	Triplets with demise of one fetus at 12 weeks—CVS performed on surviving two fetuses:
					46,XX
					47,XY,+21
**Positive trisomy 18; Y detected**	12 weeks	14.9%	0.25	0.59	Triplets with demise of one fetus—high AFP (attributed to demise)
					Remaining twins normal ultrasound

Furthermore, samples from three euploid triplet gestations and one euploid quadruplet sample were tested from the research specimens. A summary of the Y MR for these specimens, along with diagnostic outcome information is shown in Table 8.

**Table 8:** [Y Cohort: Research specimens] cfDNA and diagnostic information for triplet and quadruplet research specimens.

Fetal number	Fetal fraction of specimen	Y MR	Fetal sexes from diagnostic testing
3	10.6%	1.14	3 males
3	13.9%	0.91	2 males, 1 female
3	23.7%	0.35	1 male, 2 females
4	21.2%	0.57	2 males, 2 females

## Discussion

### Aneuploid Cohort

Risk assessment for aneuploidy in twin gestations is unique and begins with ultrasound assessment of chorionicity. Dichorionicity is present in ~80% of twin pregnancies, with monochorionic twins comprising the other ~20% [11]. Dizygotic twins typically present as dichorionic (DC), diamniotic (DA) on ultrasound evaluation, though in some cases, the placentas may appear fused. The majority (~75%) of monozygotic twins will be monochorionic (MC) and diamniotic (DA) on ultrasound, with fewer cases (~25%) presenting as DC/DA, or monochorionic (MC) and monoamniotic (MA) (<1%), depending on the timing of spontaneous embryo division. Per the American College of Obstetricians and Gynecologists, “If only one placenta is visualized, the best ultrasonographic characteristic to distinguish chorionicity is the twin peak sign [aka. the lambda or delta sign]” [12].

In general, when cfDNA screening is positive in a monochorionic twin pregnancy, the result is expected to reflect both fetuses, as the twins are presumed to have originated from the same zygote. For positive cfDNA results in a dichorionic pregnancy, there is an increased risk for aneuploidy in at least one fetus. As the majority of these pregnancies are derived from two separate zygotes, the most likely scenario is one affected twin. Less commonly, both twins may be affected if the dichorionic pregnancy was derived from a single zygote, or if both fetuses were affected as a result of independent nondisjunction events occurring in each of the dizygotic twins.

In the context of a dichorionic pregnancy with abnormal cfDNA screening results, data from massively parallel sequencing, specifically MR associated with the aneuploid chromosome, may be a useful tool for interpreting whether one or both fetuses are affected. Comparing the ‘affected fraction’ to the overall fetal fraction of the specimen may give insight into whether ‘all’ or only some of the cfDNA being contributed by the two placentas is abnormal. Though this information may assist in result interpretation and patient counseling, it should not be considered a replacement for diagnostic testing.

Based on prior studies [3, 9] and limited cases from the current study, singleton gestations affected with aneuploidy appear to be a suitable proxy for twin gestations in which both fetuses are affected with aneuploidy, either because the pregnancy is monochorionic, or because both placentas in a dichorionic gestation are impacted by the same aneuploidy. For trisomy 21, there was no significant difference noted between the MRs of affected singleton gestations and twin gestations in which both fetuses were affected.

It may be noted that a wide range of MRs are seen for the true positive singleton cases included in this study population (Table 1). As discussed in a previous publication [1], this variability may exist for several reasons. For instance, lower MRs may be associated with placental mosaicism, which appears to occur more commonly in pregnancies affected with trisomy 13 and 18, compared to trisomy 21. Biologically, this variability in MR is anticipated to impact both singleton and multifetal gestations in a similar manner, and this prediction is confirmed by the data in the current study.

For all three trisomies, the average MR of affected singletons (proxy for two affected twins) was significantly higher than the average MR of twins in which one fetus was affected (two-sided t-test, p < .001 for trisomy 21, 18, and 13, respectively). For trisomy 21, the average mosaicism ratio associated with one affected twin was 52% of that of affected singletons or cases where both twins were affected. Similarly, the MR of one affected twin was 42% of that seen in singletons for trisomy 18, and 48% for trisomy 13.

Therefore, in the rare situation where there is concern for aneuploidy in both fetuses of a dichorionic twin pair, the mosaicism ratio associated with a positive cfDNA result may be helpful in determining the probability of one versus two affected fetuses. This is expected to be an uncommon scenario, as dichorionic monozygotic twins are a rare occurrence, and independent aneuploidy events occurring in both fetuses of a dizygotic twin pair are even less frequent.

Analysis of the distribution of aneuploid MRs by trisomy found that different MR cutoffs for trisomy 21 versus trisomy 18 and 13 may be helpful in predicting whether one versus both fetuses are affected with aneuploidy in the rare situation where clinical concern arises. For trisomy 21, using an MR cutoff of 0.7 for chromosome 21 found that 77.8% (42 of 54) of twin samples with one affected fetus fell below this threshold; whereas, only 4.4% (20 of 454) of affected singleton samples had an MR <0.7. For trisomy 18 and 13, using an MR cutoff of 0.5 was found to provide the greatest separation of data. For trisomy 18, 84.2% (16 of 19) of twin samples with one affected fetus showed an MR <0.5, compared to 6.7% (12 of 179) of affected singletons. For trisomy 13, 77.8% (7 of 9) of twin samples with one affected fetus showed an MR <0.5, compared to only 4.5% (3 of 67) of affected singletons (Table 2).

### Y Cohort

The primary application of MR is in the interpretation of aneuploidy results. However, MR can also be used to analyze the relative proportion of Y chromosome material present in a multifetal gestation compared to the overall fetal fraction to determine if one or more fetuses are male when Y material is detected. Though this information may not offer widespread clinical utility, determination of fetal sex may have clinical applications for pregnancies at risk for X-linked disorders, or situations where fetal sex is ambiguous from ultrasound evaluation. Again, it should be noted that cfDNA screening results are not an acceptable substitute for diagnostic testing for the confirmation of fetal chromosomal sex, the determination of risk for sex-linked disorders, or the evaluation of disorders of sexual development.

To determine the accuracy of fetal sex classifications in twin gestations with Y chromosome material detected, Y MRs from the clinical and research specimens were analyzed. As it has been previously established that the aneuploidy status of the fetus can impact fetal fraction [8] and fetal fraction is a primary driver of mosaicism ratio, fetal sex prediction models were compared for the overall cohort (including euploid and aneuploid cases) and for euploid-only cases. Using a Y MR cutoff of 0.8 in the prediction of XX/XY versus XY/XY twin gestations is expected to have a 95.9% accuracy for euploid gestations, and a 92.3% accuracy when euploid and aneuploid gestations were combined (Tables 4 and 6).

### Triplets and quadruplets

Five cases of triplets were identified from the clinical specimens that received positive cfDNA screening results for aneuploidy, and at least partial clinical or diagnostic outcome information was provided to the laboratory. The available data, though limited, suggest that mosaicism ratio may also have clinical application to multifetal gestations beyond twins. For example, one case involved triplets that were positive for trisomy 21 with Y chromosome material detected. The MR of chromosome 21 was 0.40, and the Y MR was 0.63. Amniocentesis confirmed two male fetuses (one with trisomy 21, one euploid) and one female euploid fetus (47,XY,+21; 46,XY; 46,XX) (Table 7).

In the research cohort, three euploid triplet specimens and one euploid quadruplet specimen were analyzed (Table 8). As demonstrated with twin specimens, Y MR increased in proportion to the number of male fetuses present in the pregnancy. In combination with the clinical specimens, these data suggest that MR may have utility in interpretation of higher-order multifetal cfDNA results.

Additional studies could focus on collection of outcome information to further establish the clinical utility of MR in the context of these unique multifetal cases.

### Study limitations

Working with this retrospective cohort of multifetal gestations was not without limitations. As noted above, the laboratory does not routinely elicit information about chorionicity for multifetal specimens submitted for screening or diagnostic testing. In turn, specimens for which only one diagnostic result was available with no mention of monochorionic twins or twin-twin transfusion syndrome as an indication for testing, were excluded from analysis, as the authors were unable to discern whether testing was from a monochorionic pregnancy or from a single fetus sampled from a dichorionic gestation. Therefore, it is reasonable to assume that some number of monochorionic pregnancies were unnecessarily excluded from analysis. Furthermore, it was not possible to determine which, if any, cases were analyzed following demise of an additional fetus, which could have impact on the mosaicism ratio associated with the cfDNA screening specimen. Finally, given the study’s exclusion criteria and the biological nature of twinning and aneuploidy (making affected multifetal pregnancies relatively rare), there was variability in the number of cases available for analysis in each cohort, making it difficult to directly compare data across cohorts.

## Conclusions

Data interpretation is an essential part of cfDNA screening, and over time, laboratory bioinformatics can be leveraged to improve the accuracy of this assessment. One data metric, mosaicism ratio, has been shown to have clinical utility in refining the positive predictive value of abnormal screening results in singleton gestations. With regard to multifetal pregnancies, the same metric may be applied to determine if one or more fetuses are affected with aneuploidy, and to provide information about the possible sex of each fetus when Y chromosome material is detected. This data may help clinicians provide additional information to patients for counseling and result interpretation, but should not be considered a substitute for diagnostic testing.

## Supporting information

S1 Fig [Aneuploid Cohort: Clinical + Research specimens] distribution of mosaicism ratios for aneuploid chromosomes in affected singletons vs. one affected twin by trisomy. (PDF)

S2 Fig [Y Cohort: Clinical + Research specimens] distribution of Y chromosome mosaicism ratio in XX/XY and XY/XY twin pregnancies. (PDF)
