# Supplementary material for: Application of mosaicism ratio to multifetal gestations
Source: PLoS One. 2021 Mar 12;16(3):e0248467. doi: 10.1371/journal.pone.0248467 (PMC7954340; doi:10.1371/journal.pone.0248467)

S1 Fig: [Aneuploid Cohort: Clinical + Research Specimens] Distribution of mosaicism ratios for aneuploid chromosomes in affected singletons vs. one affected twin by trisomy.

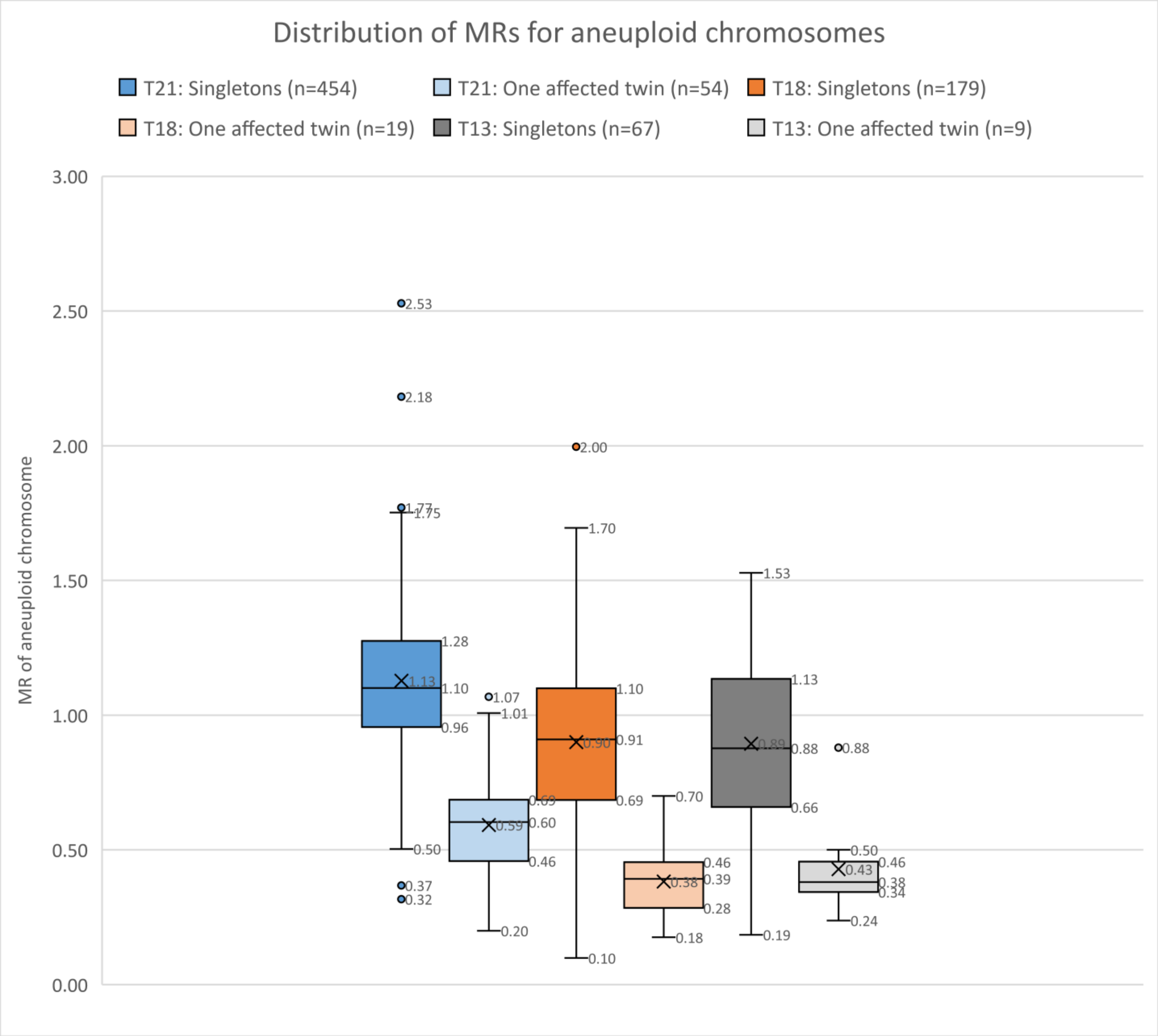

Supplement: S1 Fig — (PDF) [file pone.0248467.s001.pdf]
