# Supplementary material for: Application of mosaicism ratio to multifetal gestations
Source: PLoS One. 2021 Mar 12;16(3):e0248467. doi: 10.1371/journal.pone.0248467 (PMC7954340; doi:10.1371/journal.pone.0248467)

S2 Fig: [Y Cohort: Clinical + Research Specimens] Distribution of Y chromosome mosaicism ratio in XX/XY and XY/XY twin pregnancies.

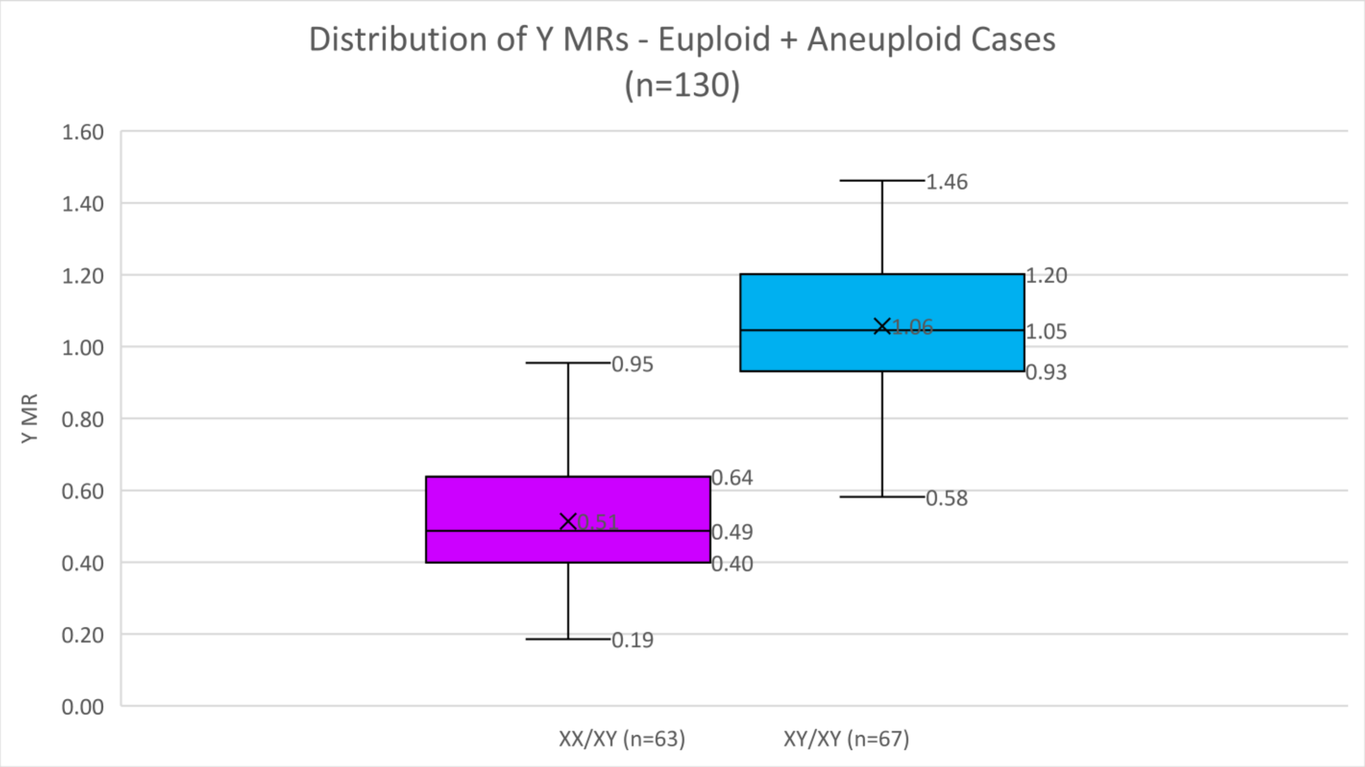

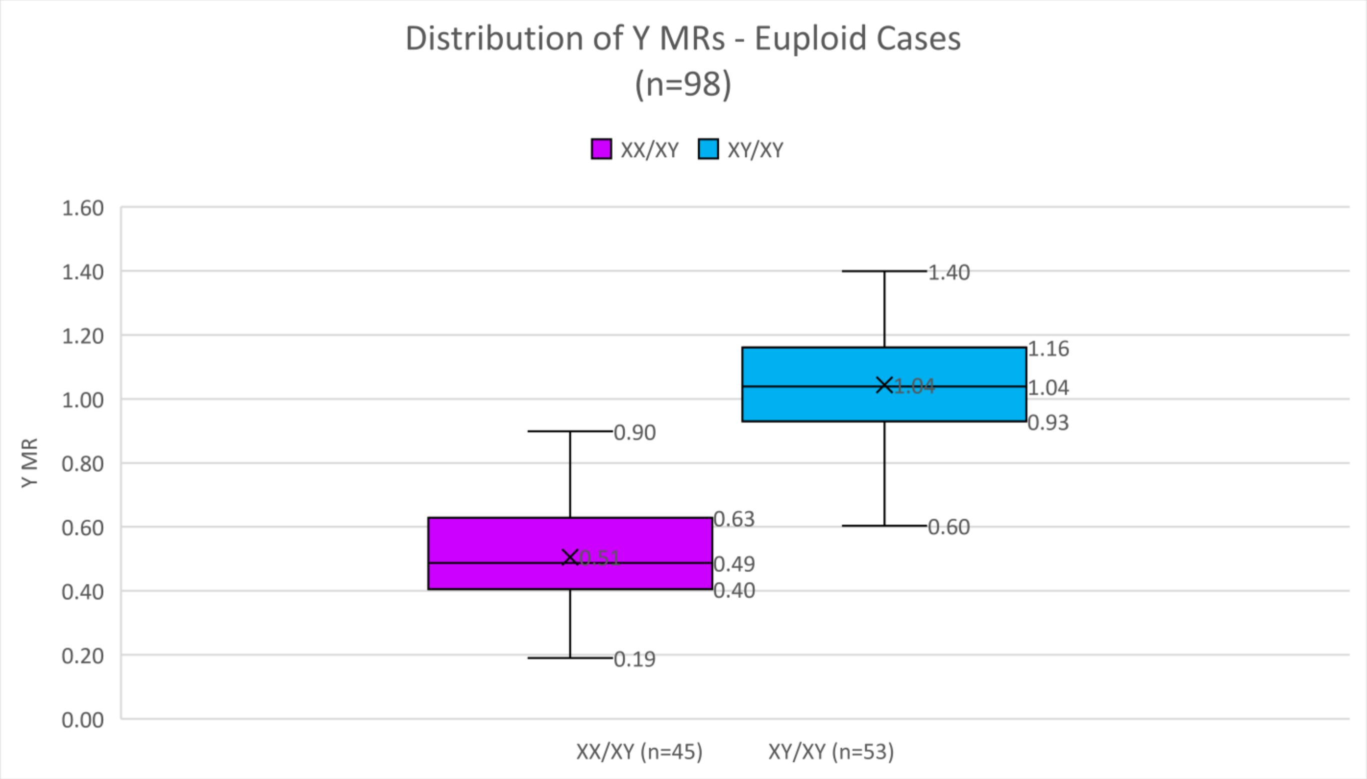

Supplement: S2 Fig — (PDF) [file pone.0248467.s002.pdf]
